# Characterization of thin film Parylene C device curvature and the formation of helices via thermoforming

**DOI:** 10.1088/1361-6439/acdc33

**Published:** 2023-07-27

**Authors:** Brianna Thielen, Ellis Meng

**Affiliations:** 1 Alfred E. Mann Department of Biomedical Engineering, Viterbi School of Engineering, University of Southern California, Los Angeles, CA, United States of America; 2 Ming Hsieh Department of Electrical and Computer Engineering, Viterbi School of Engineering, University of Southern California, Los Angeles, CA, United States of America

**Keywords:** Parylene C, thermoforming, annealing, three-dimensional, fabrication, flexible electrode array

## Abstract

In microfabricated biomedical devices, flexible, polymer substrates are becoming increasingly preferred over rigid, silicon substrates because of their ability to conform to biological tissue. Such devices, however, are fabricated in a planar configuration, which results in planar devices that do not closely match the shape of most tissues. Thermoforming, a process which can reshape thermoplastic polymers, can be used to transform flat, thin film, polymer devices with patterned metal features into complex three-dimensional (3D) geometries. This process extends the use of planar microfabrication to achieve 3D shapes which can more closely interface with the body. Common shapes include spheres, which can conform to the shape of the retina; cones, which can be used as a sheath to interface with an insertion stylet; and helices, which can be wrapped around nerves, blood vessels, muscle fibers, or be used as strain relief feature. This work characterizes the curvature of thin film Parylene C devices with patterned metal features built with varying Parylene thicknesses and processing conditions. Device curvature is caused by film stress in each Parylene and metal layer, which is characterized experimentally and by a mathematical model which estimates the effects of device geometry and processing on curvature. Using this characterization, an optimized process to thermoform thin film Parylene C devices with patterned metal features into 0.25 mm diameter helices while preventing cracking in the polymer and metal was developed.

## 1. Introduction

When implanting sensors, electrodes, or other devices into biological tissue, the use of soft polymers is preferred over rigid materials (such as silicon or metal) due to favorable mechanical properties that more closely match the physical properties of tissue. By using soft materials which can conform to surrounding tissue, the body’s immune response to implanted devices is greatly decreased (as compared to hard, rigid materials) [1].

As the field of neurological research advances, microelectromechanical systems (MEMS) devices have become invaluable as they are able to produce smaller sensors and electrodes capable of interfacing with smaller populations of neurons. Traditionally, microfabrication is used to build complex devices on a flat, rigid substrate (usually silicon), which can be assembled into three dimensional (3D) structures via stacking or linkages. Biomedical applications, however, often require more complex 3D geometries to seamlessly interface with complex anatomy. By producing MEMS devices on a flexible polymer substrate instead of a traditional silicon backbone, such devices can be transformed into 3D geometries (via post-processing) to conform to the target tissue and minimize the body’s immune response to implanted devices.

Many polymers are compatible with existing microfabrication techniques and have been used for several decades to produce a variety of flexible devices to interface with the body. The most commonly used polymers are PDMS (polydimethylsiloxane), polyimide, and Parylene C (poly(monochloro-*p*-xylylene)), all of which are significantly less stiff than silicon and more closely match the stiffness of biological tissue [2–4]. Parylene C is a thermoplastic polymer, which allows thin films to be transformed into a new 3D configuration by thermoforming against a template [1, 5]. PDMS and polyimide are thermoset polymers which can be bent into complex shapes, but cannot be softened and re-shaped after curing in a planar configuration, and require attachment to a supporting structure [6–8] or plastic deformation (which can damage the device) [9] to maintain a new, non-planar shape.

Thermoformed Parylene C has been used in numerous microfabricated medical devices [1, 10–15] with various geometries. Of note is the helix geometry, which can be used for strain relief, to interface with anatomical features such as nerves, muscle fibers, or blood vessels, or can be wrapped around cylindrical supports (such as catheters, stents, and probes) to produce 3D MEMS devices. The smallest Parylene helix reported in literature is 1.1 mm in diameter. The smallest published thermoformed Parylene feature is a 0.25 mm cylinder on a Parylene–metal–Parylene (PMP) electrode array, however features at this size exhibited cracking in both the Parylene and metal layers, rendering the device non-functional [1, 15]. This work describes a method for thermoforming Parylene helices down to 0.25 mm (a 4× improvement) while maintaining electrical conductivity in the metal layer of a PMP device, extending the design space for thin film helical devices.

## 2. Background

### 2.1. Parylene C

Parylene C is semicrystalline, having both crystalline and amorphous regions. As the material is heated above the glass transition temperature (approximately 60 °C–90 °C [1, 15, 16]), the amorphous regions will soften, allowing reorganization of the polymer chains while still in a solid state. Although Parylene is commonly used as a coating or insulation layer in electronics, in this paper we discuss Parylene-based thin film devices microfabricated in a planar, layered formation. When used with metal layers, Parylene served as a structural backbone and electrical insulation.

### 2.2. Film stress

The deposition of any thin film on a substrate produces residual stresses in the film. Film stress can either be tensile (positive), pulling the substrate into a concave ‘U’ shape, or compressive (negative), pushing the substrate into a convex shape and sometimes causing buckling or wrinkling in the thin film.

Metal thin films (which are commonly deposited via e-beam evaporation, sputtering, or electroplating) generally have high stress; however, stress can be modulated by varying deposition methods or process parameters. In most cases, sputtering produces a film with higher tensile stress than e-beam evaporation or electroplating (∼185 MPa, ∼95 MPa, and ∼10 MPa, respectively, for 700–800 nm gold thin films on silicon substrates [17, 18]). Stress in sputtered and evaporated films can be modulated by altering the pressure, power, and deposition rate (all of which impact deposition temperature, with higher temperatures leading to higher (more tensile) stress) [17–22]. Electroplated metal films, while lower stress, are usually much thicker than sputtered or evaporated films (in the range of several 100 s of nm to a few *µ*m) and require a conductive surface for deposition. In polymer-based thin film devices, a conductive surface would need to be deposited by evaporation or sputtering, negating the benefits of lower-stress electroplating [17, 18]. Thermal annealing of metal films after deposition has also been shown to increase the film stress (more tensile). In gold films, this effect is seen at temperatures of 50–100 °C or higher [17, 18], whereas platinum films require significantly higher temperatures to alter film stress (∼550–600 °C or higher) [20–22] which exceeds the processing temperature ranges for most thin film polymers.

Parylene C films have lower residual stress than metal layers (in the range of −6 MPa (compressive) to 0.3 MPa (tensile) [14, 16]) but are generally 10–100 times thicker (10’s of microns versus 100’s of nanometers). Thermal annealing (heating above the glass transition temperature) of Parylene films allows amorphous regions of the polymer to reorganize, causing increased crystallinity and shrinkage of the polymer [1, 5, 11, 16, 23–26]. These physical changes in the Parylene result in increased stress (on the order of ∼10–50 MPa), with higher annealing temperatures resulting in higher (more tensile) stress [16, 26, 27].

Although it has not been widely studied, metal films deposited on top of Parylene C films often have high compressive stress due to the thermal expansion mismatch between the materials [28]. During deposition, the metal source is heated above its melting point and transferred to the substrate, causing localized heating of the substrate at the surface where it is deposited. The coefficients of thermal expansion (CTEs) of commonly used metals are higher than that of silicon wafers (14.2 ppm for gold and 9 ppm for platinum, 3–5 ppm for silicon [29]), resulting in a higher degree of shrinkage in the metal films as compared to the silicon substrate when cooling back to room temperature, resulting in a tensile stress in the metal film [17]. The opposite is true when depositing a metal on top of Parylene; the CTEs of common metals are lower than that of Parylene C (35 ppm [30]), resulting in a higher degree of shrinkage in the Parylene layer and a compressive stress in the metal film.

### 2.3. Thin film devices

In thin film medical devices, multiple layers of polymer and metal films are stacked, resulting in unbalanced film stress between layers. When devices are released from the substrate, this can lead to curvature in the device towards the layer with the highest tensile stress (as compressive layers expand and tensile layers shrink). For dual Parylene layer devices, this curling effect is increased when asymmetric Parylene layer thicknesses are used or in areas where Parylene has been etched away (such as exposed metal areas for electrode sites or bondpads), as the stress between Parylene layers is not balanced and the high-stress metal layer is no longer on or near the neutral axis, resulting in curling towards the more tensile layer [11, 28, 31]. This curvature becomes more severe after annealing due to Parylene shrinkage (and thus stress increase) at high temperatures (up to ∼250 °C) [14, 28]. Because shrinkage of metal layers is generally observed at much higher temperatures, metal layers can buckle or wrinkle during Parylene shrinkage, resulting in changes in the patterned metal geometry. Curling can also be more pronounced when thin polymer layers are used, as they cannot overcome forces imposed by the high-stress metal layer(s).

Two processes utilize the stress and physical property changes of Parylene above the glass transition temperature for functional purposes. Annealing is commonly used to increase crosslinking between multiple Parylene layers (preventing delamination), increase crystallinity (decreasing water ingress through the material), and flatten parts which have a slight curvature when taken off the wafer. Parts are clamped between two flat surfaces, heated above the glass transition temperature while under vacuum, held for a predetermined time (in most cases, between 100 and 300 °C for 1–48 h), and cooled back to room temperature. Thermoforming is a similar process that is used to transform parts into a 3D shape. Parts are fixtured into the desired shape (rather than clamping flat), heated above the glass transition temperature, and cooled back to room temperature, at which point the polymer re-hardens and retains the fixtured shape [1, 11, 15, 23, 24, 28, 32–34].

Although thermoplastic Parylene can be annealed or thermoformed to hold a desired shape (flat or 3D), these processes can result in unexpected curvature or shape if the film stresses in a multi-layer (Parylene and metal) device are too high or not balanced in favor of the final shape. This work evaluates the impact of annealing and thermoforming process parameters on the resulting shape of bare Parylene and PMP devices. Recommendations to manage stress by varying Parylene layer thickness and annealing parameters to achieve the desired structure are discussed.

## 3. Materials and methods

### 3.1. Fabrication of bare Parylene strips

Bare Parylene strips (300 *µ*m width, 20 mm length, varying thickness; table 1) were fabricated to evaluate thermoforming capability and cracking failure. Parylene C was deposited via a chemical vapor deposition-like process (PDS 2010 Labcoter, Specialty Coating Systems, Indianapolis, IN) onto 4″ prime silicon wafers. Parylene thickness was varied from 5.4 to 20.9 *µ*m by altering the amount of Parylene dimer (Specialty Coating Systems, Indianapolis, IN) loaded into the machine. After breaking vacuum and removing the samples, an optional second Parylene C layer was added on top of the first layer in some samples (samples with a top layer thickness listed in table 1) using the same procedure. Parylene films were removed from the wafer by cutting with a scalpel and peeling the cut region off the wafer. The film was then cut into strips by mounting on an adhesive mat and trimming to the final shape using a cutting plotter (Graphtec CE6000-40, Irvine, CA).

**Table 1.** Parylene C thicknesses for bare Parylene strip samples. Samples with no top layer thickness listed were constructed of a single Parylene layer.

Parylene thickness (*µ*m)		
Base layer	Top layer	Total
5.6	n/a	5.6
9.9	n/a	9.9
5.5	5.6	11.1
13.3	n/a	13.3
5.3	13.2	18.5
20.9	n/a	20.9
5.4	20.4	25.8
14.4	11.9	26.3

### 3.2. Fabrication of PMP devices

**Figure 1.**
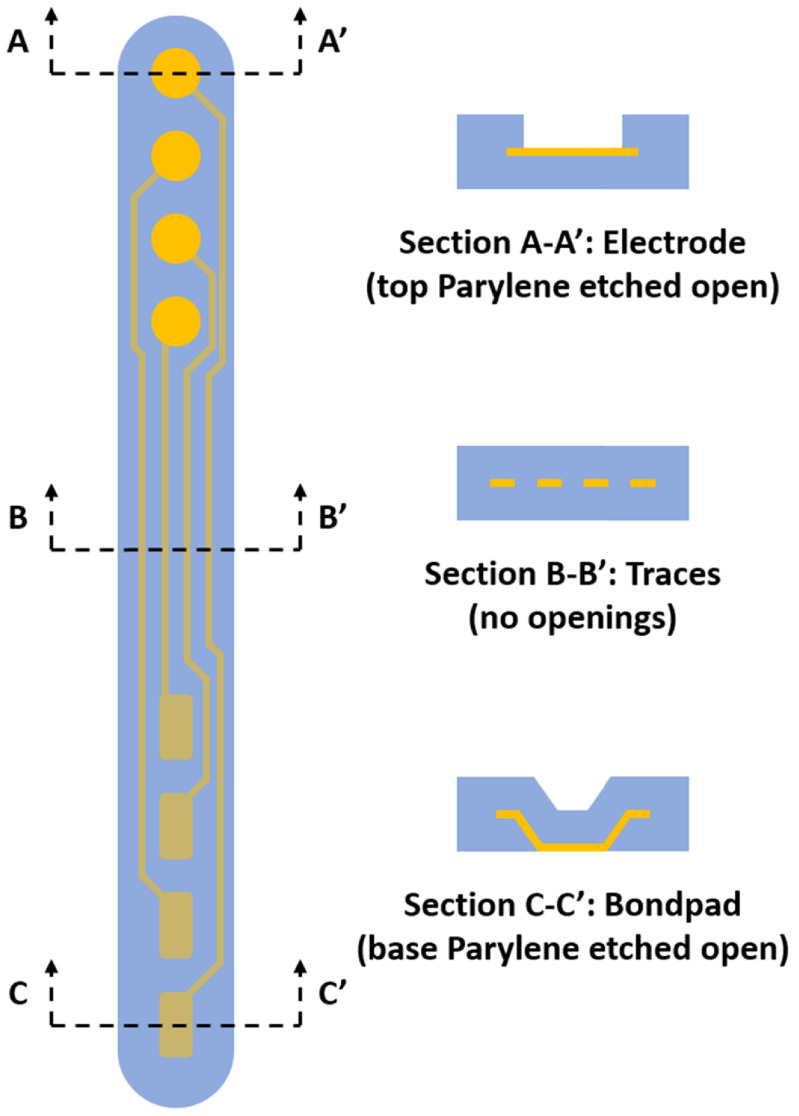
Diagram of a Parylene–metal–Parylene device configuration, with openings etched in the top Parylene to form electrodes (section A-A′), insulated traces (section B-B′), and openings etched in the base Parylene to form bondpads (section C-C′).

**Figure 2.**
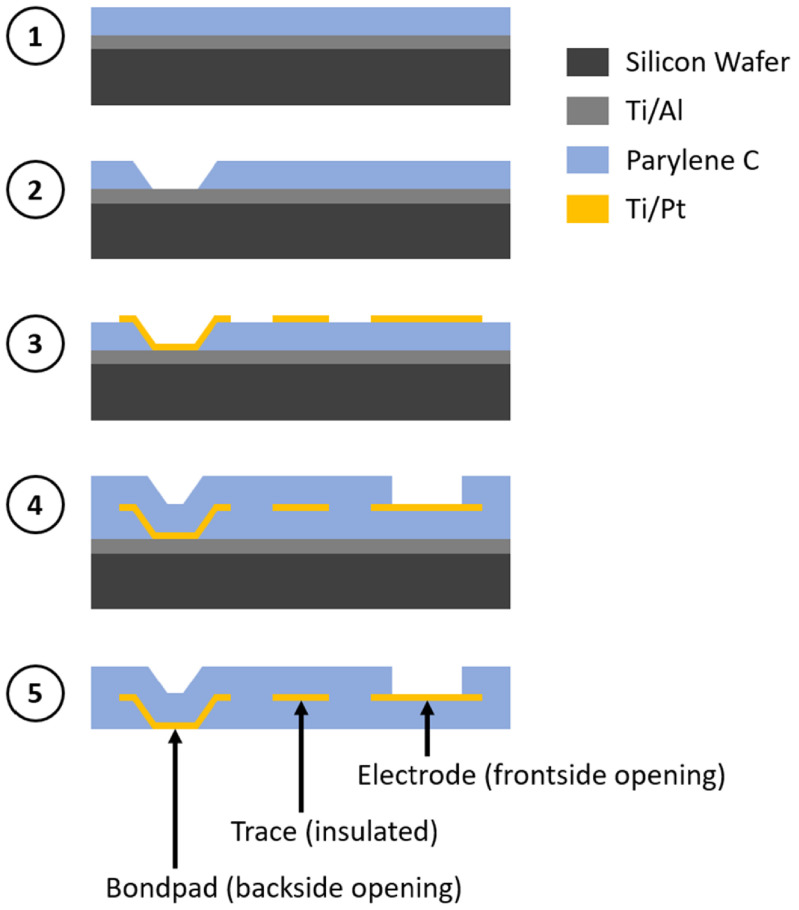
Cross-sectional view of Parylene–metal–Parylene device fabrication process flow. (1) Parylene was deposited on top of a sacrificial aluminum layer (with titanium adhesion layer; Ti/Al). (2) Backside bondpads were opened via O_2_ etch. (3) Platinum (with titanium adhesion layer; Ti/Pt) electrodes, traces, and bondpads were deposited and patterned. (4) Parylene was deposited on top of patterned metal and frontside electrodes were opened via O_2_ etch. (5) The aluminum layer was dissolved and devices were released.

PMP electrode arrays were fabricated to evaluate device curling as a result of residual stress and test thermoforming parameters on realistic device configurations. The PMP devices consisted of one metal layer (platinum) sandwiched between two Parylene C layers, with openings in the Parylene C to expose electrode sites on the frontside of the device on one end and bondpads on the backside of the device on the opposite end. A diagram of a representative PMP device is shown in figure 1, and the fabrication process is summarized in figure 2.

Two groups of devices with varying dimensions were fabricated (thick/asymmetric and thin/symmetric devices—dimensions for each group are listed in table 2) to evaluate different design parameters such as the total thickness, the impact of asymmetric Parylene layers, and the impact of pre-annealing the base Parylene layer. A minimum layer thickness of ∼3 *µ*m was chosen to prevent device damage due to handling, and a maximum total thickness of ∼15 *µ*m was chosen to maintain device flexibility and to prevent excessive etching times.

**Table 2.** Dimensions for Parylene-metal-Parylene electrode arrays.

Group	Parylene thickness (*µ*m)			Device width (*µ*m)	Device length (mm)	Number of traces	Trace width (*µ*m)
	Base layer	Top layer	Total				
Thick/Asymmetric	3.4	11.5	14.9	350	20 + 50^a^	16	5–10
Thin/Symmetric	4.4^b^	4.7	9.1	210	40	8	5

^a^ Thick/Asymmetric devices were ‘L’ shaped, with one 20 mm arm and one 50 mm arm.

^b^ The 4.4 *µ*m base Parylene layer in group B was annealed at 150 °C for 4 h before adding metal or top Parylene layers.

PMP devices were fabricated using a low temperature, batch process based on prior work [35]. 4′′ prime silicon wafers were coated with a 10 nm titanium adhesion layer followed by 100 nm of aluminum, which was chemically roughened by etching for 8 min in CR-7 etchant (Transene, Danvers, MA) to promote Parylene C adhesion to the surface. The Parylene C base layer was deposited using a chemical vapor deposition-like process (PDS 2010 Labcoter, Specialty Coating Systems, Indianapolis, IN) to a thickness of either 3.4 or 4.4 *µ*m (see table 2). Openings in the Parylene for metal bondpads on the backside of the device were etched using O_2_ reactive ion etching (PlasmaPro 80 RIE, Oxford Instruments, Bristol, UK; 150 mT, 150 W, 50 sccm O_2_; etch rate approximately 0.2 *µ*m min^−1^) masked by patterned photoresist (AZ P4620, AZ Electronic Materials, Branchburg, NJ; 12 *µ*m thick; reflowed at 110 °C for 20 s to produce angled sidewalls). After etching, photoresist was removed using acetone followed by rinsing in isopropyl alcohol and deioinized water.

For the thin/symmetric device group only, the coated wafers were baked (annealed) in an oven (TVO-2, Cascade Tek Inc., Longmont, CO) under vacuum with nitrogen flow at 150 °C for 4 h. Next, lithography was performed to add a lift-off photoresist mask (AZ 5214 IR, AZ Electronic Materials, Branchburg, NJ; 1.8 *µ*m thick). Then, a 15 nm titanium adhesion layer and 200 nm platinum layer were deposited via e-beam evaporation. Electrodes, traces, and bondpads were formed following lift-off in 40 °C acetone followed by rinsing in isopropyl alcohol and deioinized water. After metal patterning, a top layer of Parylene C was deposited to a thickness of either 11.5 or 4.7 *µ*m (see table 2). Openings in the Parylene for metal electrodes on the frontside of the device were etched using O_2_ reactive ion etching (150 mT, 150 W, 50 sccm O2; etch rate approximately 0.2 *µ*m min^−1^) or O_2_ switched chemistry etching in a deep reactive ion etcher [36] (PlasmaPro 80 or PlasmaLab 100 ICP, respectively, Oxford Instruments, Bristol, UK; switched chemistry process parameters detailed in [36]; etch rate approximately 0.08 *µ*m/loop) masked by patterned photoresist (AZ P4620; 8–12 *µ*m thick), and the outline of the device was cut out using the same etching procedure. Photoresist was removed using acetone after each etching step followed by rinsing in isopropyl alcohol and deioinized water. Devices were released from the wafer by dissolving the aluminum adhesion layer in AZ MIF 726 (AZ Electronic Materials, Branchburg, NJ) at 60 °C.

### 3.3. Thermoforming and annealing

After cutting bare Parylene strips to shape or releasing PMP devices from the wafer, the parts were annealed flat or thermoformed into helices to evaluate the effects of heat treatment on device shape and failure modes.

To fixture parts for annealing, the parts were placed between two Teflon sheets (0.03 mm thick) and clamped flat between two glass slides using clips.

**Figure 3.**
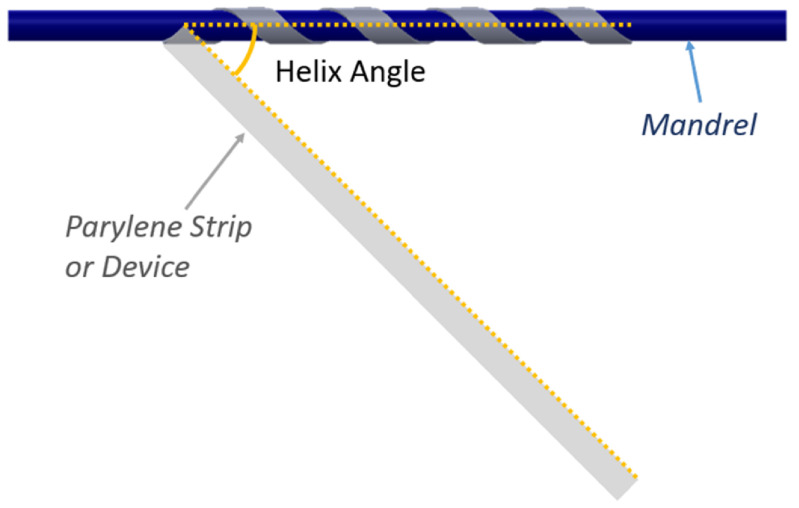
Illustration of the helix angle—the angle between the axis of the mandrel and the long edge of the Parylene strip or device (helix angles of 15°, 30°, and 45° were used).

To fixture parts for thermoforming, the parts were wrapped around a stainless steel mandrel of the desired diameter into a helical shape by hand using a template to define the helix angle (see figure 3) and held in place using Teflon film (0.01 mm thick).

After fixturing, the parts were placed into a programmable vacuum oven (TVO-2, Cascade Tek Inc., Longmont, CO), placed under vacuum, then purged three times with nitrogen to minimize oxygen in the chamber. The oven was programmed to ramp up to a defined temperature at a ramp rate of approximately 0.7 °C min^−1^, annealed for a defined time, then ramp down to room temperature. After cooling, parts were removed from the fixture and inspected.

### 3.4. Thermoforming tests: bare Parylene strips

Bare Parylene strips (table 2) were thermoformed into helices (2.6, 1.6, and 0.25 mm helix diameters; 15°, 30°, and 45° helix angles) following the procedure listed in section 3.3 with a thermoforming temperature of 200 °C and hold time of 12 h. Select samples were repeated at thermoforming temperatures of 150 and 100 °C. Double-layer Parylene strips were tested in both winding directions (i.e. two samples were run, one with the base layer towards the inside of the helix, and one with the top layer towards the inside of the helix) to evaluate the effects of uneven Parylene layers.

After thermoforming, the Parylene helices were visually inspected using a microscope (HD60T, Caltex Scientific, Irvine, CA and Eclipse LV100, Nikon, Tokyo, Japan) for their ability to retain the desired shape and for any defects (cracking) in the Parylene.

### 3.5. Annealing tests: bare Parylene strips

Bare Parylene strips (table 2) were annealed flat following the procedure listed in section 3.3 with a thermoforming temperature of 200 °C and hold time of 12 h to determine if bare Parylene films (single or double layer) experienced any curling due to film stress without a metal interposing layer.

After annealing, the Parylene strips were visually inspected and photographed using a microscope (HD60T, Caltex Scientific, Irvine, CA).

### 3.6. Annealing tests: PMP devices

PMP devices (table 3) were annealed flat following the procedure listed in section 3.3 with variable temperature and hold time. Due to the high number of variables (temperature, hold time, Parylene layer thicknesses, base layer annealing), two representative groups were chosen to qualitatively evaluate several variables experimentally while corroborating with published literature.

**Table 3.** Thermoforming result vs. thickness, helix angle, and helix diameter for bare Parylene strips at 200 °C thermoforming temperature and 12 h thermoforming time. ✓ indicates a good result, $*$ indicates minor cracking (partial-thickness), × indicates cracking (full-thickness), and • indicates loose shape. Only parts indicated with a † were tested at a 30° helix angle; all parts were tested at 15° and 45°. Parts indicated with a ‡ were also tested at 100 and 150 °C, yielding the same results.

			30°, 45° helix angle				15° helix angle			
Parylene thickness (*µ*m)			Helix diameter (mm)				Helix diameter (mm)			
Inner layer	Outer layer	Total	2.6	1.6	0.25		2.6	1.6	0.25	
5.6	n/a	5.6^†^	✓	✓	✓		✓	✓	✓	
9.9	n/a	9.9^†^	✓	✓	$*$		•	✓	$*$	
5.5	5.6	11.1	✓	✓	$*$		•	•	✓	
13.3	n/a	13.3^‡^	✓	✓	×		✓	✓	×	
5.3	13.2	18.5^†‡^	✓	✓	×		•	•	×	
13.2	5.3	18.5^†‡^	✓	✓	×		•	✓	×	•
20.9	n/a	20.9^†^	✓	✓	×		•	✓	×	
5.4	20.4	25.8	✓	✓	×	•	✓	✓	×	•
20.4	5.4	25.8	✓	✓	×		✓	✓	$*$	•
11.9	14.4	26.3^†^	✓	✓	×		✓	✓	×	•
14.4	11.9	26.3^†^	✓	✓	×		•	•	×	

For all parts, the curvature of the PMP devices before and after any annealing treatment was measured by photographing the parts using a microscope (HD60T, Caltex Scientific, Irvine, CA) and measuring the radius of curvature using edge detection in MATLAB.

#### 3.6.1. Effects of base layer annealing.

Devices from each sample group were inspected prior to full device annealing to evaluate the effects of base layer annealing (which was done for thin/symmetric samples, but not thick/asymmetric samples). Although devices from each group did not have equal thickness or symmetry, results were supported in comparison to similar processes found in literature and by the mathematical model described in section 4.

#### 3.6.2. Effects of asymmetric Parylene layers.

Devices from each sample group were annealed (full annealing experiments described in sections 3.6.3 and 3.6.4) to evaluate the impact of asymmetric Parylene layers (present in the thick/asymmetric group, but not the thin/symmetric group). In addition, the bondpad and electrode regions (with local areas of Parylene removed in the base and top layers, respectively) were evaluated separately to determine the effects of exposed metal on resulting device curvature. Although devices from each group did not have equal thickness and the thin/symmetric group had a base layer annealing step, results were supported by results from similar processes found in literature and by the mathematical model described in section 4.

#### 3.6.3. Effects of annealing time.

Devices from each sample group were annealed for 0.5, 6, 12, 24, or 48 h at an annealing temperature of 200 °C. The curvature diameters of devices before and after annealing were compared to determine the effects of annealing time on Parylene shrinkage (and thus overall device curvature) and to determine the amount of time necessary to reach the maximum shrinkage. A 200 °C annealing temperature was chosen as it is a common annealing temperature used for Parylene in other published devices and experiments [15, 24, 28, 32, 33, 37].

#### 3.6.4. Effects of annealing temperature.

Devices from each sample group were annealed for 12 h at an annealing temperature of 100, 150, or 200 °C. The curvature diameters of devices before and after annealing were compared to determine the effects of annealing temperature on Parylene shrinkage (and thus overall device curvature) and magnitude of shrinkage at each temperature. A 12 h annealing time was selected as it was found to be sufficient to achieve maximum shrinkage in the annealing time experiments.

### 3.7. Thermoforming tests: PMP devices

Based on results from the bare Parylene strip thermoforming tests and PMP device annealing tests, PMP devices were thermoformed using both the thermoforming parameters used in the bare Parylene thermoforming experiments (200 °C for 12 h) and optimized parameters determined from PMP annealing experiments. For each sample group, thermoforming temperature and time were selected which yielded devices closest to the target curvature in the PMP device annealing experiments, keeping layer direction in mind (i.e. if devices were curled towards the base layer after annealing, the base layer was wound to the inside of the helix; if devices were curled towards the top layer after annealing, the top layer was wound to the inside of the helix). Several sets of parameters (low and high temperatures) were tested for each sample group.

After thermoforming, the PMP devices were visually inspected using a microscope (HD60T, Caltex Scientific, Irvine, CA and Eclipse LV100, Nikon, Tokyo, Japan) for their ability to retain the desired shape and for cracking in the Parylene. PMP devices were also electrically tested for continuity between the bondpads and electrodes using an LCR meter (E4980A, Agilent Technologies, Santa Clara, CA) before and after thermoforming. The LCR meter was used to measure the impedance between each bondpad/electrode pair using a 10 kHz, 20 mV signal. Traces were considered continuous if the impedance magnitude was less than 100 kΩ and phase was greater than −65°.

## 4. Freed film curvature modeling

Stress in thin films has been extensively studied and modeled, with most studies focusing on a single material deposited on a rigid surface [38, 39]. Although this configuration differs from that of thin-film biomedical devices, which consist of multi-layer, freed thin films, similar analysis can be used to mathematically model such devices. In addition, film stress measurements on rigid substrates can be used to predict the curvature of freed, multi-layer films. Although the devices used in this study (and in most thin film biomedical devices) have complex geometries (patterned Parylene and metal layers), a simplified model structure can be used to predict the behavior of thin film devices using several fundamental equations describing the stress and curvature in composite structures. Such a model can be used to predict the curvature of fabricated devices if other device parameters (dimensions and stress) are known.

**Figure 4.**
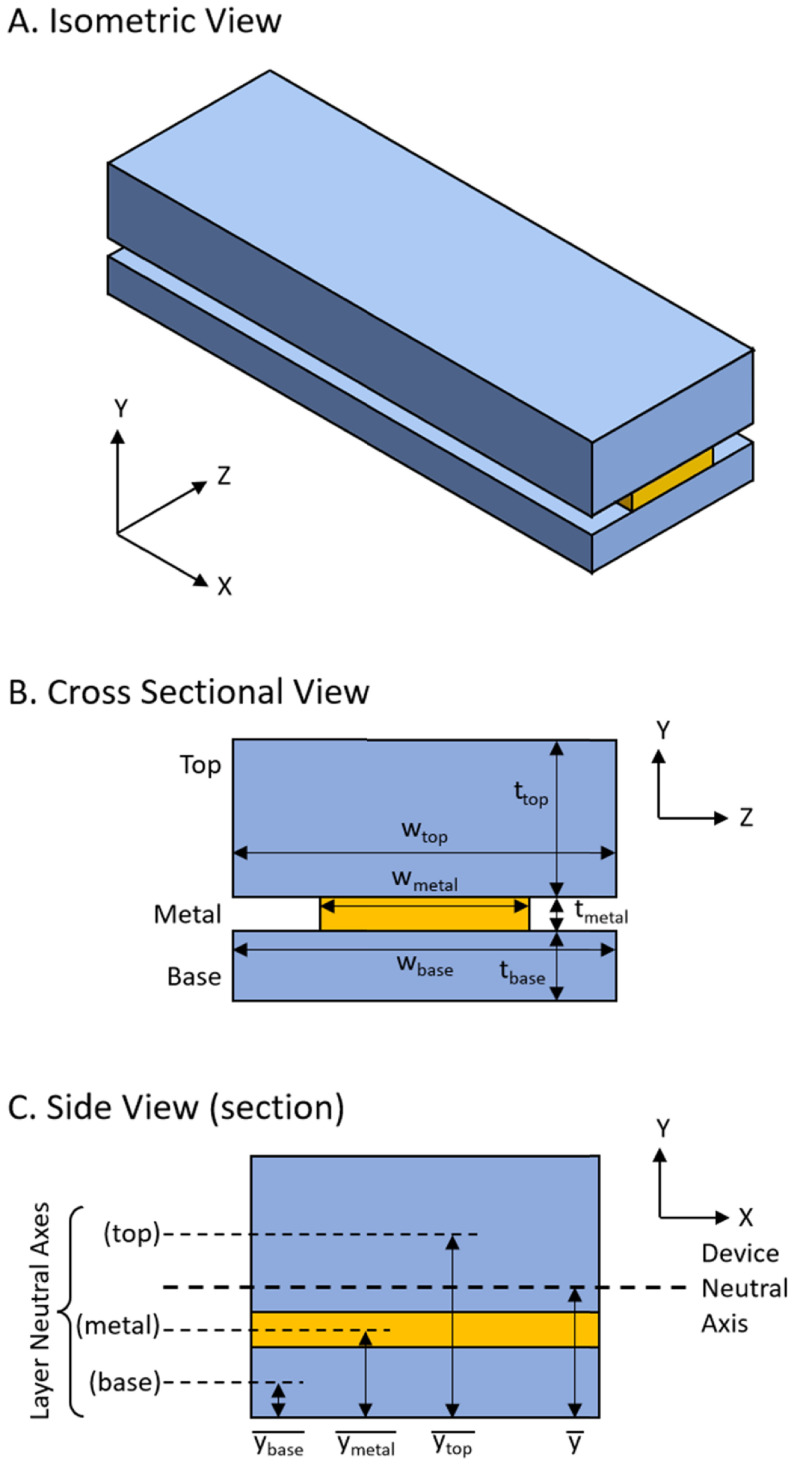
(A) An isometric view and (B) a cross sectional view of the simplified model geometry, with a single metal strip sandwiched between two Parylene layers. (C) The side view shows a small section of the device with the neutral axis of each layer and the full device.

Figure 4 shows a simplified thin film device consisting of a base Parylene layer, a metal layer, and a top Parylene layer, modeled as a composite I-beam. This model simplifies the trace region of the device shown in figure 1 section B-B′ by combining the individual traces into one metal film (which should not impact the device curvature about the *Z* axis if the overall metal width and thickness are identical) and ignores any effects due to contact between the base and top Parylene layers. The model can also be used to simulate the electrode or bondpad regions by altering the width of the top or base layers to account for the smaller amount of Parylene in that area.

**Figure 5.**
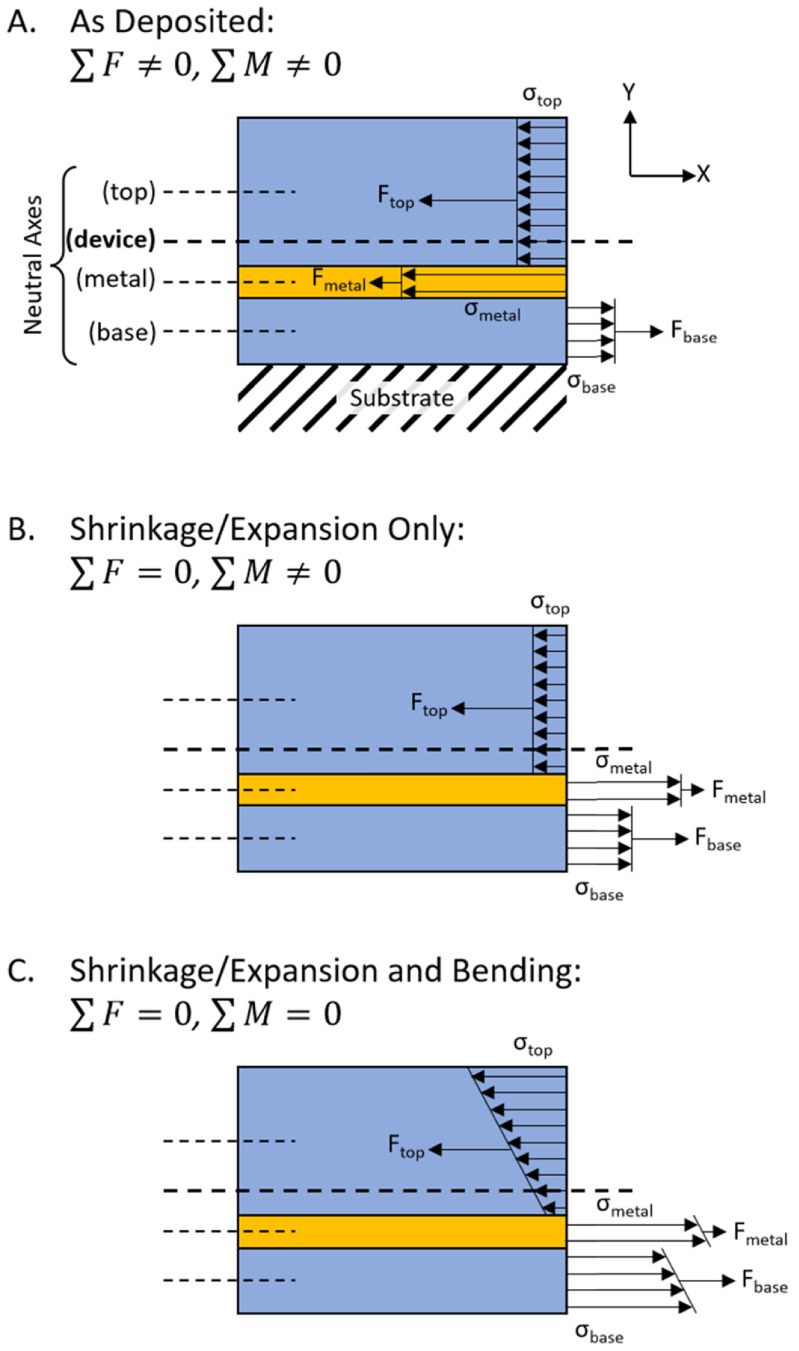
Illustration of the stresses and forces acting on a section of the device (side view, as shown in figure 4(C)). (A) As deposited: each film layer is deposited on a substrate with residual stress, producing a part with stresses leading to unbalanced force and moment (about the neutral axis). (B) Shrinkage or expansion only: When removed from the substrate, the part shrinks or expands, adjusting the stresses in each layer to balance the forces in the device. (C) Shrinkage/expansion and bending: After shrinking or expanding, the device curls to balance the moment about the neutral axis, resulting in a stress gradient in each layer. *Note:* the metal layer illustrated here is not to scale, so the high stress leads to a small force, and the stress changes significantly in each step.

Figure 5(A) shows a small section of the device and the stresses and forces acting along one edge of that section before any stresses have been allowed to balance (i.e. before the device has been released from the wafer). In the case shown in the figure, the base Parylene layer has a moderate tensile stress (${\sigma _{{\text{base}}}}$), the middle metal (platinum) layer has a large compressive stress (${\sigma _{{\text{metal}}}}$), and the top Parylene layer has a small compressive stress (${\sigma _{{\text{top}}}}$). The stresses in each layer can be measured or estimated from literature (common stresses in deposited Parylene and platinum films found in literature are included later in this section). These stresses can be converted into forces using the relationship

\begin{align*}{F_i} = {\sigma _i}{A_i}\end{align*}

where ${F_i}$ is the force and ${A_i}$ is the cross-sectional area (width, ${w_i}$ times thickness, ${t_i}$), and the subscript $i$ represents each layer (i.e. base Parylene, metal, or top Parylene). When the device is released from the wafer, the device will either compress or expand to equalize the forces in each layer (figure 5(B) shows the device having expanded slightly due to the net compressive force in figure 5(A)), resulting in a net force of zero.

This is described mathematically as

\begin{align*} {{\mathop \sum \nolimits }}{{}}F_i^{^{\prime}} &amp; = 0 = F_{{\text{base}}}^{^{\prime}} + F_{{\text{metal}}}^{^{\prime}} + F_{{\text{top}}}^{^{\prime}} \nonumber\\ &amp; = \sigma _{{\text{base}}}^{^{\prime}}{A_{{\text{base}}}} + \sigma _{{\text{metal}}}^{^{\prime}}{A_{{\text{metal}}}} + \sigma _{{\text{top}}}^{^{\prime}}{A_{{\text{top}}}} \end{align*}

where $F_i^{^{\prime}}$ and $\sigma _i^{^{\prime}}$ are the forces and stresses in each layer after shrinkage or expansion. Because each layer is bonded to the adjacent layer, the overall strain in each layer is equal. Using this relationship, the stress before and after expansion in each layer are described as

\begin{align*}\varepsilon = \frac{{\sigma _{{\text{base}}}^{^{\prime}} - {\sigma _{{\text{base}}}}}}{{{E_{{\text{Pa}}}}}} = \frac{{\sigma _{{\text{metal}}}^{^{\prime}} - {\sigma _{{\text{metal}}}}}}{{{E_{{\text{metal}}}}}} = \frac{{\sigma _{{\text{top}}}^{^{\prime}} - {\sigma _{{\text{top}}}}}}{{{E_{{\text{Pa}}}}}}\end{align*}

where $\varepsilon $ is the strain and ${E_k}$ is the Young’s modulus of the material for that layer (note that the relationship in equation (3) assumes no delamination between films, resulting in equal strain in every layer; Pa is Parylene). Using equations (2) and (3), $\sigma _i^{^{\prime}}$ for each layer can be calculated and converted into $F_i^{^{\prime}}$ using equation (1).

Although the forces in the device are now balanced, the forces are not acting at the neutral axis of the device, resulting in a net moment. The net moment in the device is

\begin{align*}M = \mathop \sum \nolimits {M_i} = \mathop \sum \nolimits F_i^{^{\prime}}\left( {\overline {{y_i}} - \bar y} \right)\end{align*}

where $M$ is the net moment in the device, ${M_i}$ is the moment in each layer, $\overline {{y_i}} $ is the neutral axis of each layer, and $\bar y$ is the neutral axis of the device. The resulting moment (due to the expanded, balanced forces) is what causes the device to curl. The bending moment equation describes the radius as

\begin{align*}r = \frac{{EI}}{M}\end{align*}

where $r$ is the radius of curvature and $I$ is the second moment of area of the device. Note that, in order to use this equation, the equivalent area method must be used when calculating $I$ to represent the full device as a single material with Young’s modulus $E$. The second moment of area for each component about the device neutral axis, ${I_i}$, is

\begin{align*}{I_i} = \frac{{{w_i}t_i^3}}{{12}} + {A_i}{\left( {\overline {{y_i}} - \bar y} \right)^2}\end{align*}

which can be used to calculate the device second moment of area, $I$, as

\begin{align*}I = {I_{{\text{base}}}} + \frac{{{E_{\textrm{metal}}}}}{{{E_{{\text{Pa}}}}}}{I_{\textrm{metal}}} + {I_s}\end{align*}

which represents the entire device as being made from Parylene using the equivalent area method.

The released, curled device has an adjusted stress gradient in each layer (due to the shrinkage or expansion and curvature of the device), resulting in both net zero force and moment about the neutral axis (figure 5(C)).

Each of the testing conditions used in this study for PMP device annealing (described in section 3.6) were evaluated using the model with similar dimensions to the experimental device geometries (width and thickness of each layer). Deposited film stress and Young’s modulus for each material were found in literature or determined by fitting experimental data to the model.

The stress in Parylene films has been shown to increase with higher annealing temperature. While all published data referenced for this study follows this trend, exact stress values over the given temperature range (20–200 °C) differ between publications [16, 26, 27]. In addition, films in these studies were annealed in air while attached to a substrate. When exposed to oxygen at high temperatures, Parylene will oxidize, impacting the resulting mechanical properties of the material [1, 24]. In this study, PMP device annealing was performed after devices had been released from their silicon carrier wafer and clamped between two flat surfaces (as described in section 3.3) in a nitrogen-purged vacuum oven. The difference in annealing fixturing likely resulted in different stress changes and resulting device shape because the devices can shift slightly during the annealing process.

No data has been published on the stress in platinum films deposited via e-beam evaporation on Parylene, however qualitative descriptions estimate it to be less compressive than sputtered platinum on Parylene (with a value of −511 MPa) as evidenced by less wrinkling in the metal layer and less curvature in released devices [28, 31]. Modeled Parylene and platinum stress were selected by sweeping the Parylene stress within the bounds of the published data and the platinum stress from −511 to 0 MPa and selecting values which most accurately matched the results of the PMP device annealing experiments (section 5.3). This resulted in Parylene stress of −3.46 MPa at 20 °C (as deposited) linearly increasing 0.16 MPa/°C and platinum stress of −100 MPa. No published work has described the changes in Parylene stress with varying annealing time, so the variable annealing time condition evaluated in this study (section 5.3.3) could not be modeled.

Due to these differences, modeled results differ slightly from experimental data under some conditions but follow a similar trend. Where applicable, the model was applied to the experimental data to produce a direct comparison (described in section 5.3). The model was also used to illustrate general trends in PMP device shape with different annealing conditions and device dimensions (with results included in supplementary information S1). These trends support experimental results found in section 5.3 and can be used to motivate future PMP device designs.

## 5. Experimental results

### 5.1. Thermoforming tests: bare Parylene strips

**Figure 6.**
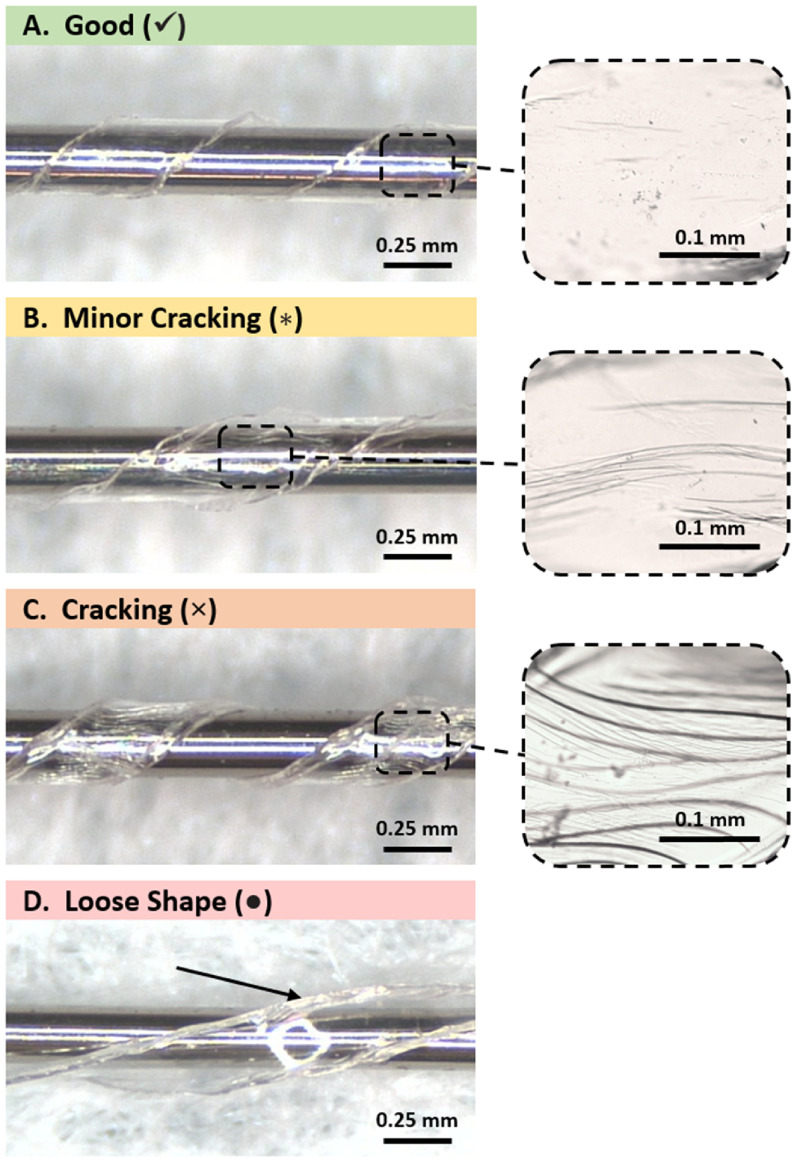
Examples of bare Parylene strips (300 *µ*m width by 20 mm length, variable thickness) thermoformed into 0.25 mm helices, showing a good result (✓), minor cracking ($*$), cracking (**×**), and loose shape (•).

When fixturing bare Parylene strips into helices and thermoforming, no cracking was observed for any Parylene thickness tested when thermoforming to a 1.6 or 2.6 mm helix diameter at any helix angle (example of a bare Parylene helix with no cracking is shown figure 6(A)). At 0.25 mm diameter, helices with thinner Parylene (⩽11.1 *µ*m total thickness) exhibited minimal cracking (figure 6(B); partial-thickness cracking) or no cracking. Thicker films (⩾13.3 *µ*m total thickness) exhibited cracking (figure 6(C); through the full thickness of the Parylene) at the same diameter due to the increased bending stress experienced by thicker samples. Cracking in the Parylene appeared after fixturing around the mandrel and prior to thermoforming, indicating that the cracks were formed during the fixturing process due to the stress induced during bending.

The thickness of the Parylene and the helix diameter had no apparent impact on the resulting shape after thermoforming. All parts (except for a single outlier) held the desired shape when wrapped at a 45° helix angle. When parts were wrapped at a 15° angle, several parts did not retain the desired shape after thermoforming (figure 6(D)), however there is no trend in the shape failures, indicating a fixturing problem for these parts. Parylene strips which were tested at a 30° helix angle had identical results to parts of equivalent thickness with a 45° angle (however not all thicknesses were tested at 30°).

Parylene strips constructed of two layers had comparable results wrapped in either direction (i.e. with the base layer towards the inside of the helix or the top layer towards the inside of the helix). Although the residual stress in deposited Parylene films has been reported to vary with the thickness of the film in some cases [14], these stress differences are minor and do not appear to impact the thermoforming process when a bare Parylene sample is used. PMP parts (discussed in sections 5.3 and 5.4) are more impacted by asymmetric Parylene layers due to the high stress, interposing metal layer.

Thermoforming results for bare Parylene strips are summarized in table 3, with representative examples of a good result, cracking failure, and shape failure shown in figure 6.

### 5.2. Annealing tests: bare Parylene strips

Annealing bare Parylene strips resulted in flat parts (matching the flat, fixtured shape) for all Parylene thicknesses tested (5.6–26.3 *µ*m). The parts had relatively low stress prior to annealing due to the low stress of deposited Parylene and absence of a high-stress metal layer. Parts made from a single Parylene layer were not expected to curl as the curling is a result of mismatched stress and shrinkage in parts with multiple thin film layers. Parts made from two or more thin film layers experienced curling when there was a stress mismatch between layers, however because both layers in these parts were made of the same material, the similar stress and shrinkage resulted in a flat part.

### 5.3. Annealing tests: PMP devices

Mismatched stress between the three distinct layers in PMP devices (base Parylene, metal, and top Parylene) caused devices to curl based on the net stress in the device (with resulting curvature towards highly tensile layers and away from highly compressive layers). Annealing PMP devices alters the stress in each layer, changing the magnitude and/or direction of device curling. Other parameters, such as pre-annealing the base Parylene layer or depositing asymmetric Parylene layers (among many other design and processing parameters not evaluated in this work), also impact the resulting curvature of the device. The impacts of base layer annealing, asymmetric Parylene layers, annealing temperature, and annealing time were evaluated experimentally and compared with the mathematical model.

#### 5.3.1. Base layer annealing.

**Figure 7.**
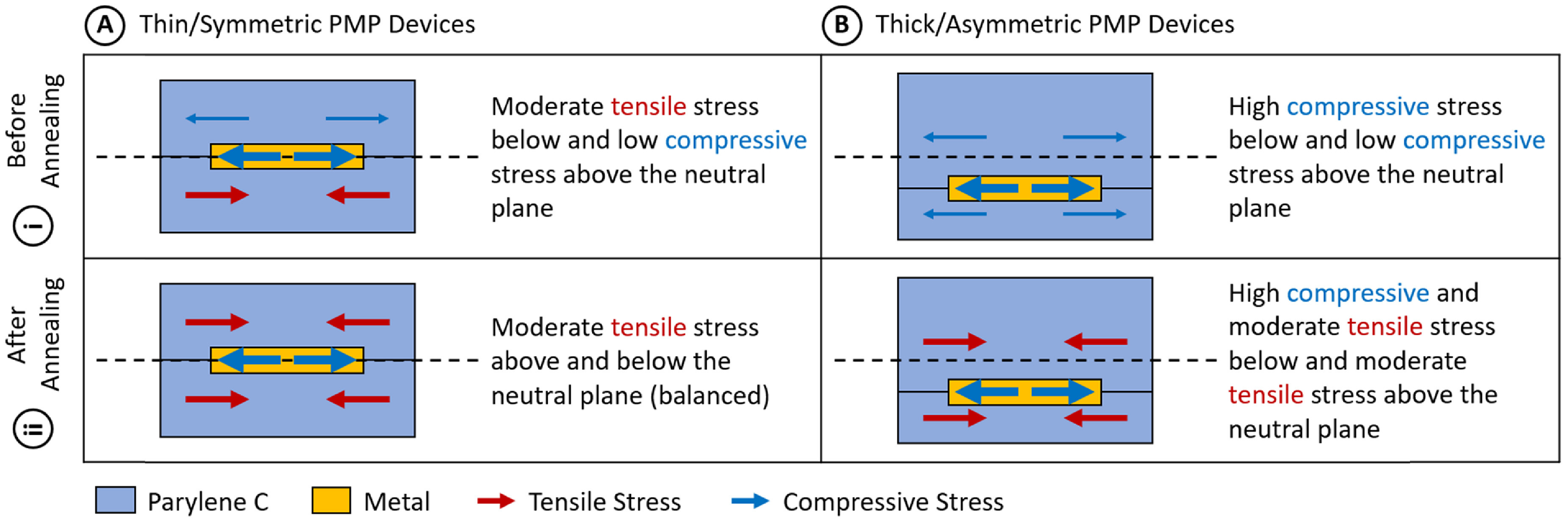
Illustration of the stress in each layer of PMP devices before and after annealing. (Ai) Thin/symmetric devices before annealing have moderate tensile stress in the base Parylene layer due to shrinkage during the base layer anneal and a low compressive stress in the top Parylene layer, resulting in a device curled towards the base layer. (Aii) Thin/symmetric devices after annealing have balanced stress around the neutral plane due to equal shrinkage in the Parylene layers and the high-stress metal layer sitting on the neutral plane, resulting in a flat device. (Bi) Thick/asymmetric devices before annealing have high compressive stress in the metal layer and low compressive stress in both Parylene layers. The compressive stress in all layers results in enough expansion in the device to produce a low tensile stress in the metal layer (below the neutral plane), resulting in a device with mild curvature towards the base layer. (Bii) Thick/asymmetric devices after annealing have high compressive stress in the metal layer below the neutral plane and equal tensile stress in both Parylene layers, resulting in a device curled towards the top (thicker) Parylene layer.

The base Parylene layer on parts in the thin/symmetric group was annealed (150 °C, 4 h) prior to addition of the metal or top Parylene layers. As a result, the stress in the base Parylene layer became more tensile (due to the shrinkage of the Parylene—see figure 7(Ai)). When thin/symmetric parts were taken off the silicon carrier wafer, they were curled towards the pre-annealed base layer to a diameter of 4.2 ± 0.6 mm (mean ± standard deviation). This experimental value is slightly larger than the value calculated by the mathematical model with equivalent conditions (2 mm towards the base layer), likely due to the differences in processing conditions when calculating Parylene stress in literature (see discussion in section 4).

Parts in the thick/asymmetric group, which did not undergo high temperature annealing prior to removal from the wafer, were curled towards the base layer to a much lesser extent (60 ± 22 mm diameter); this closely matches the value produced by the model (62 mm towards the base layer). The mild curling was due to the asymmetry of the Parylene layers in the device (described in detail in section 5.3.2).

**Figure 8.**
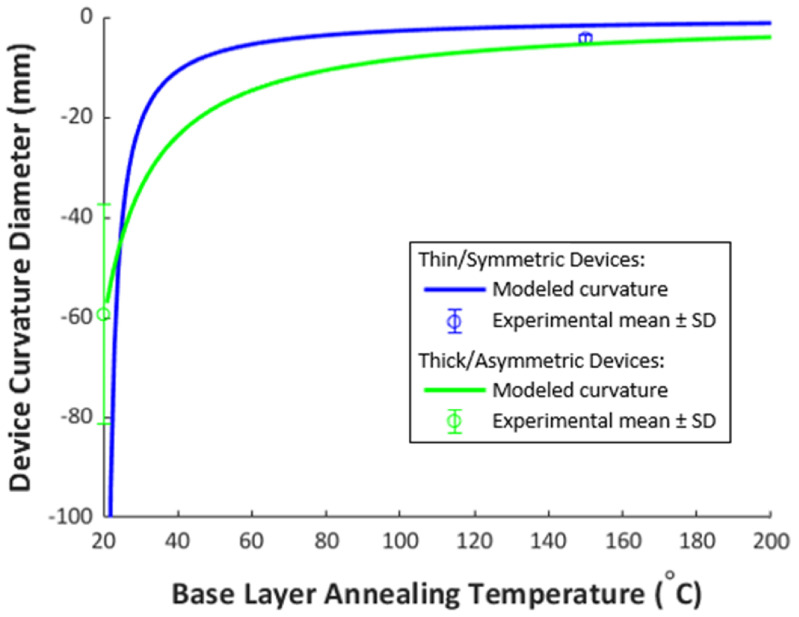
Modeled and experimental curvature diameter versus base layer annealing temperature for PMP devices. The mean value of experimental data is plotted with error bars showing one standard deviation (SD). Modeled parameters were identical to PMP device parameters for each device group (table 2). Stress was calculated from temperature based on fitting literature values to all data (described in section 4), with *σ*_top_ calculated at 20 °C (representing no anneal).

Although the Parylene thicknesses were different in the two experimental groups so a quantitative comparison cannot be made, the data suggest pre-annealing is largely responsible for the greater curling in thin/symmetric group PMP device based on similarities to other devices in literature [11, 40] and the results of the mathematical model (experimental data and the mathematical model for both device groups are plotted in figure 8).

It is also important to consider the impact of device processing conditions on the resulting Parylene and metal stress. PMP device fabrication in this study included four photoresist patterning steps, each of which required a baking step to harden the photoresist and/or for image reversal (for metal lift-off patterning only). In total, the base Parylene layer experienced temperatures of 90 °C for 6 min and 110 °C for 45 s, and the full device (base and top Parylene layers) experienced a temperature of 90 °C for 10 min. The metal deposition and device release processes also exposed the base Parylene layer and full device, respectively, to increased temperatures, however these processes did not exceed 60 °C with the equipment and parameters used in this study.

Although heating Parylene to temperatures near or above the glass transition temperature is known to cause changes in stress [16, 26, 27], it is unknown how significant these changes are during such a short heat exposure. The shortest published Parylene annealing test annealed Parylene at 50 °C for 15 min, followed by 100 °C for 15 min and found a stress increase from −6 to 17 MPa [16]; no shorter duration annealing tests have been reported to our knowledge.

#### 5.3.2. Asymmetric Parylene layers.

Asymmetry impacts the curvature of the device by moving the high stress metal layer away from the neutral plane of the device. This results in unbalanced stress about the neutral axis, producing a net moment on the device that causes it to curl. The magnitude of curling depends on the thickness of each layer (and the resulting distance between the metal layer and the neutral plane) and the processing conditions. Curling is generally most severe in annealed devices due to the increased tensile stress in the Parylene layers acting in combination with the high compressive stress in the metal layer. In unannealed devices (with no base layer anneal), every film layer has residual compressive stress (figure 7(Bi)), causing the device to expand and balance some forces prior to curling. This results in less severe curling than in an annealed device, where the tensile Parylene layers and compressive metal layer are acting against each other, resulting in a smaller degree of shrinkage or expansion and higher forces in each layer.

**Figure 9.**
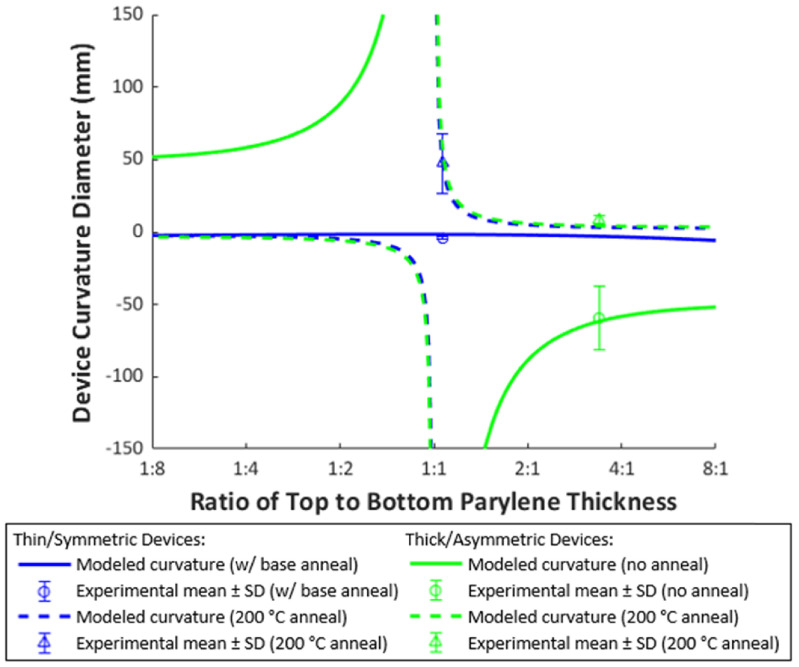
Modeled and experimental curvature diameter versus ratio of top to base layer thickness for PMP devices before and after 200 °C anneal. The mean value of experimental data is plotted with error bars showing one standard deviation (SD). Modeled parameters were identical to PMP device parameters for each device group (table 2). Stress was calculated from temperature based on fitting literature values to all data (described in section 4).

After annealing, thick/asymmetric devices followed the trend reported in literature and described by the model, curling significantly towards the thicker, top Parylene layer (down to a minimum post-annealing curvature diameter of 8.1 ± 2 mm when annealed for 12 h at 200 °C) due to comparable shrinkage in the Parylene layers and the compressive metal layer acting below the neutral plane. Thin/symmetric devices became flatter after annealing (up to a maximum post-annealing curvature diameter of 1300 mm when annealed for 12 h at 100 °C). These changes are due to the stress increase in Parylene after annealing resulting in unbalanced or balanced stress in PMP device layers. The curvature estimated by the model with equivalent parameters for thick/asymmetric devices (with or without 200 °C anneal) and thin/symmetric devices (with 200 °C anneal) closely matches the experimental values. For thin/symmetric devices with only a 150 °C base anneal, the modeled curvature value is slightly smaller than the experimental value (2 mm and 4.2 mm, respectively), likely due to the differences in processing conditions when calculating Parylene stress in literature (see discussion in section 4). Experimental data and modeled curvature for both device groups before and after annealing at 200 °C are plotted in figure 9. Detailed post-annealing curvature analysis is included in sections 5.3.3 and 5.3.4.

**Figure 10.**
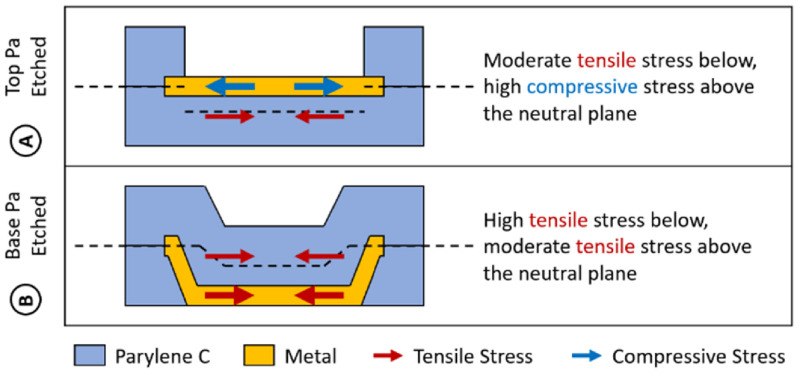
Illustration of the stress in each layer of annealed PMP devices in regions with etched Parylene openings. The neutral plane shifts in etched areas, resulting in different stress balance in each area. (A) Regions with top Parylene etched openings (electrodes) have moderate tensile stress in the base Parylene (below the neutral plane) and high compressive stress in the metal layer (above the neutral plane), resulting in curling towards the base layer. (B) Regions with base Parylene etched openings (bondpads) have moderate tensile stress in the top Parylene layer (above the neutral plane) and high tensile stress in the metal layer (due to deposition directly on silicon, not Parylene; below the neutral plane), resulting in curling towards the base layer.

In most areas on PMP devices, the top and base Parylene layers act to balance the stress in the metal layer. In small areas where Parylene has been etched away (i.e. to produce exposed metal electrodes or bondpads (see figure 1)), only two film layers are present (one Parylene, one metal), resulting in a shift in the neutral plane of the device and unbalanced stress. When the top Parylene layer is etched away, the neutral plane shifts down below the metal surface, allowing the high compressive stress in the metal layer to curl the device towards the base layer (figure 10(A)). Stronger curvature in areas with the top Parylene etched away is commonly reported in literature [1, 11, 28].

When the base Parylene layer is etched away, the metal is deposited directly on the substrate (resulting in tensile stress, instead of compressive stress when it is deposited on top of Parylene) and the top Parylene layer deposits in a conformal layer on top of the metal. This also results in the shift of the neutral plane towards the base, but with a tensile metal layer at the bottom surface of the device. This produces regions with high tensile stress in the metal layer below the neutral plane (pulling the device towards the base layer) and tensile stress in the Parylene layer above the neutral plane (pulling the device towards the top layer), as illustrated in figure 10(B). The resulting curvature depends on the thickness of the Parylene layer and the relative size of the local etched areas as compared to the full device area. In the thin/symmetric group, the Parylene was not sufficiently thick to resist the tensile stress of the metal, resulting in curvature towards the base layer. In the thick/asymmetric group, the thick top Parylene produced a stronger force, resulting in curling towards the top layer.

**Figure 11.**
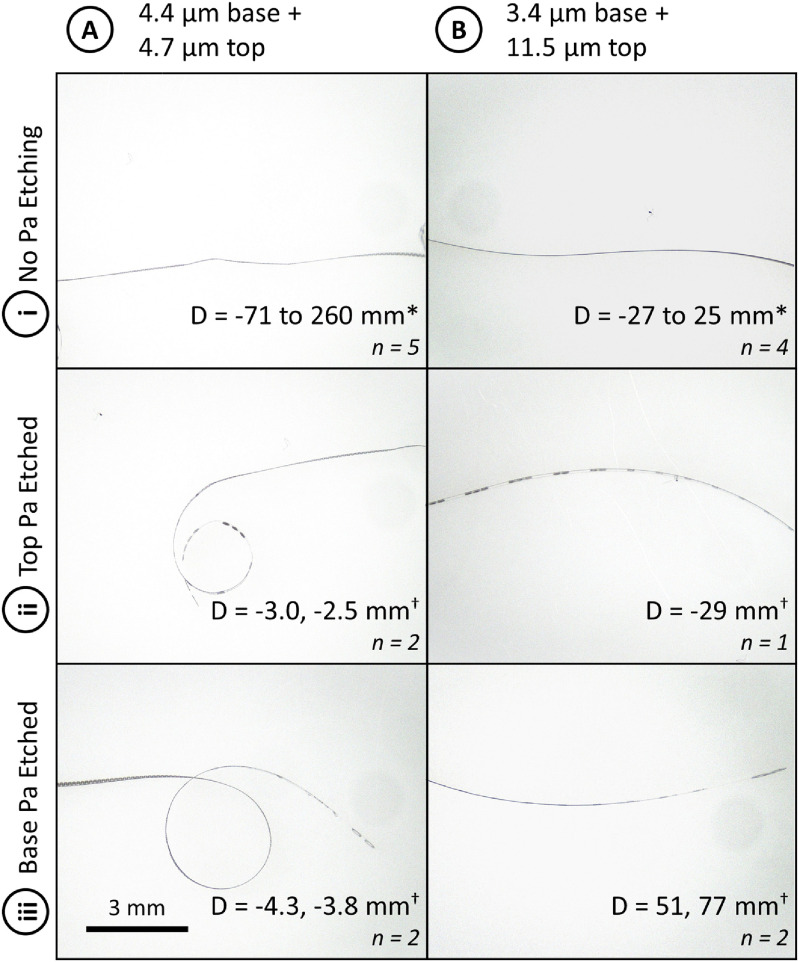
Representative photos of PMP devices annealed at 100 °C for 12 h. Column (A) shows thin/symmetric devices (4.4 + 4.7 *µ*m thickness) and (B) shows thick/asymmetric devices (3.4 + 11.5 *µ*m thickness). Row (i) shows the region of the device with no etched openings, (ii) shows the region of the device with etched openings in the top Parylene layer (electrodes), and (iii) shows the region of the device with etched openings in the base Parylene layer (bondpads). Scale bar for all photos (bottom left corner) is 3 mm; the measured average diameter (mean ± standard deviation) and number of samples measured are shown in the bottom right corner of each image. *Indicated devices curled towards both the base and top Parylene layers in different regions/samples, so the minimum diameter in each direction is included (the average diameter does not capture the physical shape because a flat device has an infinite diameter). ^†^No standard deviation is included due to insufficient sample size; measured values are listed.

**Figure 12.**
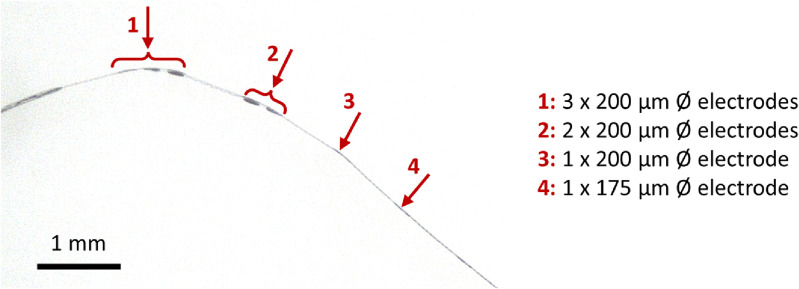
Photo of the electrode region of a thin/symmetric PMP device annealed at 200 °C for 12 h. Local regions with etched top Parylene (marked with red arrows) are curved towards the base layer, while regions between the etched openings (with a symmetric Parylene-metal–Parylene stack) are noticeably flatter.

These curvature changes in etched areas are most pronounced in devices which have been annealed at low temperature (etched and non-etched regions of devices after annealing at 100 °C for 12 h are shown in figure 11; full dataset included in supplementary information S2). In addition, only the local region with etched Parylene is affected (see figure 12), however these small areas can impact the overall shape of the device.

#### 5.3.3. Annealing time.

When annealing PMP devices, most stress changes in the films occur relatively quickly. Although a 48 h annealing time is often used to improve adhesion between Parylene layers [23, 32], the resulting curvature after annealing is similar for all tested annealing times (30 min to 48 h).

In the thin/symmetric group, devices released off the wafer (prior to annealing) had a tight curvature towards the base layer (−4.3 ± 0.7 mm diameter) and became significantly flatter after annealing (21–76 mm diameter towards the top layer when annealed at 200 °C). Differences in annealing time did not have a significant impact on resulting device curvature.

In the thick/asymmetric group, devices released off the wafer (prior to annealing) had a mild curvature towards the base layer (−59 ± 22 mm diameter) and became curled towards the top layer after annealing (4.8–23 mm diameter when annealed at 200 °C). Devices annealed for 30 min and 6 h resulted in similar curvature diameters that were less severe (larger diameter) than devices annealed for 12 h and 48 h.

**Figure 13.**
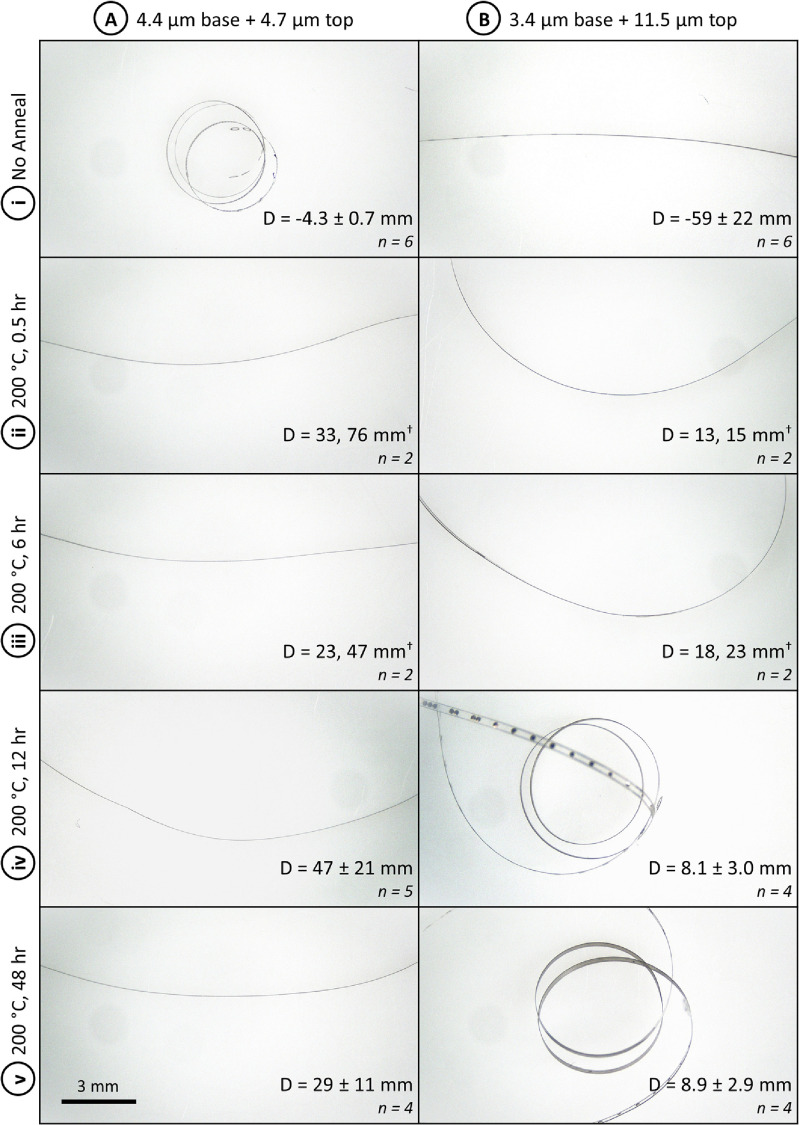
Representative photos of PMP devices annealed at 200 °C for varying times. Column (A) shows thin/symmetric devices (4.4 + 4.7 *µ*m thickness) and (B) shows thick/asymmetric devices (3.4 + 11.5 *µ*m thickness). Each row represents a different annealing time—(i) before annealing, (ii) 0.5, (iii) 6, (iv) 12, and (v) 48 h. Scale bar for all photos (bottom left corner) is 3 mm; the measured average diameter (mean ± standard deviation) and number of samples measured are shown in the bottom right corner of each image. ^†^No standard deviation is included due to insufficient sample size; measured values are listed.

Detailed curvature data and representative photos for both groups are included in figure 13 (full dataset included in supplementary information S2). The annealing time condition could not be modeled due to insufficient data available in literature.

#### 5.3.4. Annealing temperature.

Annealing temperature is shown in literature to have a significant impact on film stress and can thus be used to tailor the resulting device curvature after annealing or thermoforming, as is shown in the modeling results in the supplemental information S1. When annealing Parylene, higher temperature produces more shrinkage (due to increased tensile stress in the film), as evidenced by more significant curvature changes in PMP devices annealed at higher temperatures.

In the thin/symmetric group, devices released off the wafer (prior to annealing) had a tight curvature towards the base layer (−4.3 ± 0.7 mm diameter—due to the 150 °C base layer anneal) and became very flat after annealing at 100 °C, with mild curvature in both directions among various regions on a single device. Curvature diameters ranged from −71 mm (towards the base layer) to 260 mm (towards the top layer). As annealing temperature increased to 150 and 200 °C, the resulting parts had mild curvature towards the top, likely due to the slightly thicker top Parylene layer.

In the thick/asymmetric group, devices released off the wafer (prior to annealing) had a mild curvature towards the base layer (−59 ± 22 mm diameter) and similarly showed a flatter shape after a 100 °C anneal with mild curvature in both directions at different regions. Curvature diameters ranged from −27 mm (towards the base layer) to 25 mm (towards the top layer). As annealing temperature increased to 150 and 200 °C, the resulting parts became curled towards the top due to the significantly thicker top Parylene layer.

**Figure 14.**
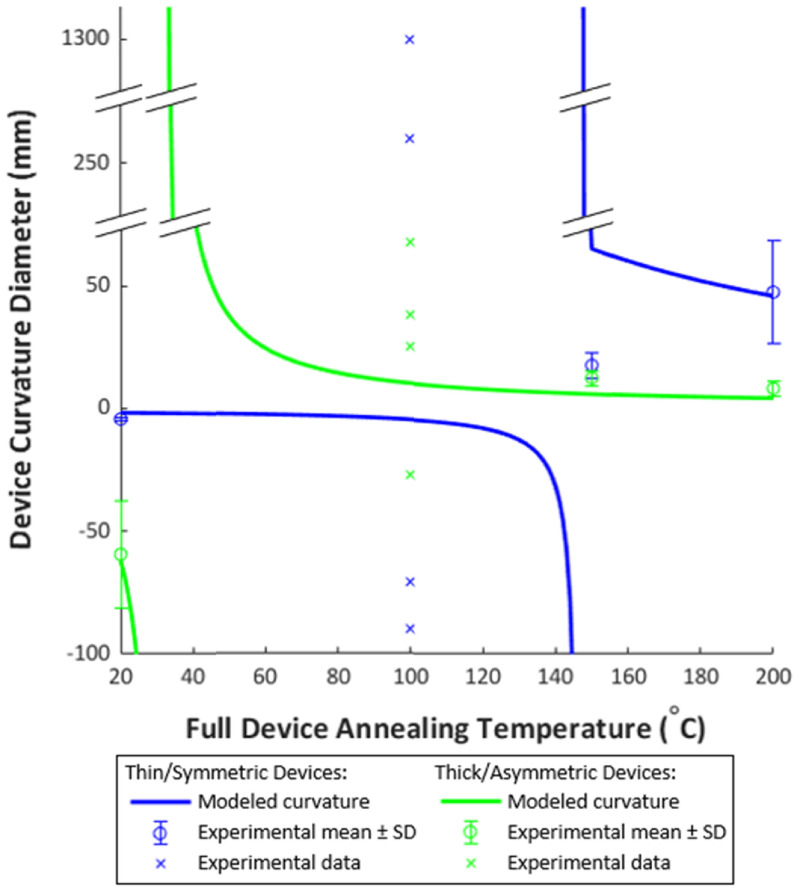
Modeled and experimental curvature diameter versus full device annealing temperature for PMP devices. The mean value of experimental data (where available) is plotted with error bars showing one standard deviation (SD). When the mean could not be calculated, all data points are plotted with an ‘x’. Modeled parameters were identical to PMP device parameters for each device group (table 2). Stress was calculated from temperature based on fitting literature values to all data (described in section 4).

In both device groups, the experimental data closely matched the mathematical model in the unannealed condition and after annealing at 200 °C, as shown in figure 14. Parts annealed at 100 and 150 °C differed significantly from the modeled curvature, to a greater extent in the thin/symmetric group. This suggests that there may be a dynamic relationship between Parylene stress and annealing temperature around the glass transition temperature that is not captured in the linear stress models published in literature. In addition, some of the fabrication conditions (which occur in the range of 60–110 °C on short time scales) may be impacting Parylene stress in ways that are not captured in the model.

**Figure 15.**
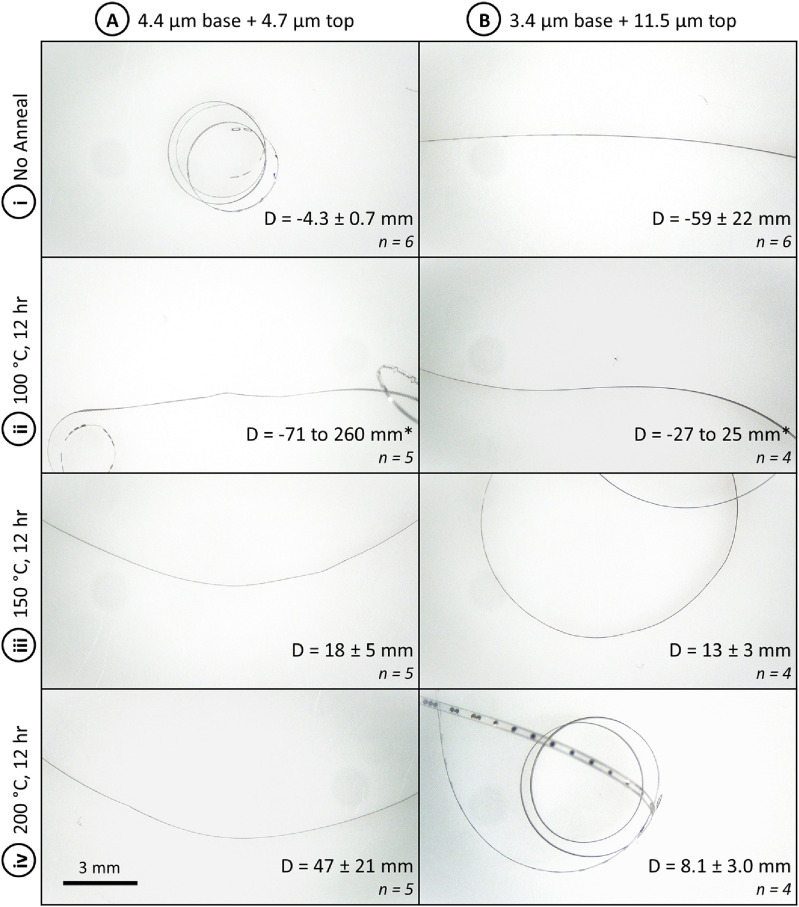
Representative photos of PMP devices annealed at varying temperatures for 12 h. Column (A) shows thin/symmetric devices (4.4 + 4.7 *µ*m thickness) and (B) shows thick/asymmetric devices (3.4 + 11.5 *µ*m thickness). Each row represents a different annealing temperature—(i) before annealing, (ii) 100 °C, (iii) 150 °C, and (iv) 200 °C. Scale bar for all photos (bottom left corner) is 3 mm; the measured average diameter (mean ± standard deviation) and number of samples measured are shown in the bottom right corner of each image. *Indicated devices curled towards both the base and top Parylene layers in different regions/samples, so the minimum diameter in each direction is included (the average diameter does not capture the physical shape because a flat device has an infinite diameter).

In both groups, the curvature of annealed devices differed significantly from unannealed devices within the group. Detailed curvature data and representative photos for both groups are included in figure 15 (full dataset included in supplementary information S2).

### 5.4. Thermoforming tests: PMP devices

**Figure 16.**
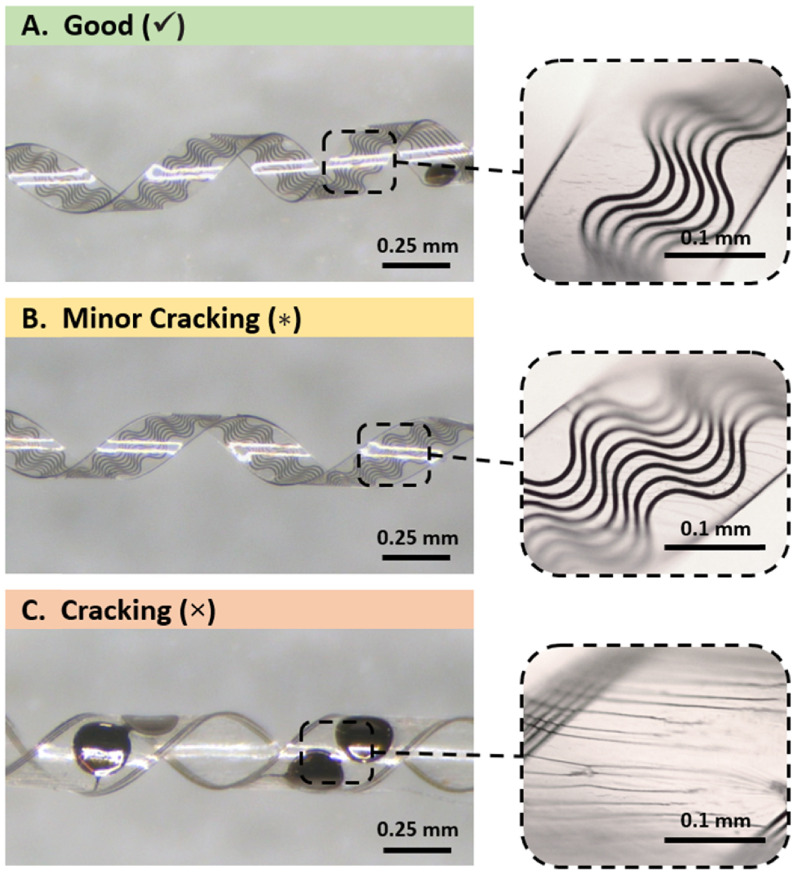
Examples of PMP devices (210–350 *µ*m width by 20–40 mm length, variable thickness) thermoformed into 0.25 mm helices, showing a good result (✓), minor cracking ($*$), cracking (×), and loose shape (•).

As was observed with bare Parylene strips, thermoformed PMP devices did not have any Parylene cracking when thermoforming to a 1.6 mm helix diameter at a 45° helix angle (figure 16(A)), with the exception of a single outlier (one sample in the thick/asymmetric group with the 11.5 *µ*m layer on the inside of the helix had minor cracking in some areas). All parts thermoformed to a 1.6 mm diameter maintained electrical conductivity (i.e. continuous traces) after thermoforming.

At 0.25 mm diameter, thin/symmetric PMP devices with the 4.4 *µ*m pre-annealed layer on the inside of the helix showed minor cracking (figure 16(B)) in some small areas and no cracking in remaining areas. These samples were the only parts to maintain electrical conductivity after thermoforming to a 0.25 mm diameter. When the 4.7 *µ*m layer was formed to the inside of the helix, minor cracking was present in all areas and metal traces did not remain continuous after thermoforming. In all thick/asymmetric samples formed to a 0.25 mm diameter helix, Parylene cracking (figure 16(C)) was observed and metal traces were not continuous.

Three configurations in the thin/symmetric group did not maintain the correct shape in the electrode region of the device after thermoforming to a 1.6 mm helix diameter (4.4 *µ*m layer on the inside of the helix thermoformed at 200 °C, and 4.7 *µ*m layer on the inside of the helix thermoformed at 100 or 200 °C). In the flat annealing tests, the electrode region of PMP devices retained a different shape (with a more significant curl away from the exposed metal surface) than the rest of the device due to high, unbalanced film stress. In thermoformed thin/symmetric devices, this imbalance led to an unexpected (loose) shape in the electrode region of most devices. In thick/asymmetric devices (all helix diameters) and thin/symmetric devices at 0.25 mm helix diameter, no loose shape was observed. Parts also maintained their shape after thermal cycling to 85 °C for 3 + hours up to 8 times over the course of 2–4 weeks.

Thermoforming results for PMP devices are summarized in table 4, with representative examples of a good result, cracking failure, and shape failure shown in figure 16.

**Table 4.** Thermoforming result vs. thickness and helix diameter for PMP devices at 45° helix angle, 200 °C thermoforming temperature, and 12 h thermoforming time. ✓ indicates a good result, $*$ indicates minor cracking (partial-thickness), × indicates cracking (full-thickness), • indicates loose shape, and—indicates discontinuous traces.

Parylene thickness (*µ*m)			Flat anneal^a^	Thermoforming temperature (°C)	Helix diameter (mm)			
Inner layer	Outer layer	Total			1.6		0.25	
4.4^b^	4.7	9.1	No	100	✓		✓	$*$ ^d^
				200	✓	•^c^	✓	$*$ ^d^
4.7	4.4^b^	9.1	No	100	✓	•^c^	$*$	—
				200	✓	•^c^	$*$	—
			Yes	200	✓		$*$	—
3.4	11.5	14.9	No	200	✓		×	—
11.5	3.4	14.9	No	200	✓		×	—
			Yes	200	✓	$*$ ^d^	×	—

^a^ The indicated parts were annealed flat at 200 °C for 48 h prior to thermoforming.

^b^ The 4.4 *µ*m base Parylene layer was deposited and annealed (150 °C, 4 h) before adding metal and top Parylene.

^c^ Most regions of the indicated parts formed to the desired shape, however some areas in the electrode region (with etched openings in the top, 4.7 *µ*m Parylene) did not retain the desired shape.

^d^ Most regions of the indicated parts had no cracking, however some minor cracking was visible in a few areas.

## 6. Discussion

The results presented in this work describe a variety of parameters that can be optimized to produce sub-millimeter helices by thermoforming Parylene thin film devices. While linear relationships between parameters cannot be established due to the large number of parameters and limited values tested for each parameter, each of the individual results from sections 5.1 through 5.3 and the variety of parameter sets used in section 5.4 and summarized in table 4 point to an optimal set of design and process parameters for producing 0.25 mm diameter helices.

Total Parylene thickness must be less than or equal to 11.1 *µ*m to prevent cracking, with even thinner layers (down to 5.6 *µ*m) performing better. This is an important consideration in medical devices, as the Parylene usually acts as both a structural backbone and an electrical insulator and must remain intact to prevent device damage and electrical leakage in the device. In addition, the optimization of bare Parylene thermoforming provides insight that could inform the design of thermoformed microfluidic channels, although additional testing would be necessary to ensure channels remained open and to characterize any dimensional changes after thermoforming. Any cracking in Parylene microfluidic channels would provide a pathway for fluid to escape the channel, rendering the device ineffective.

The base Parylene layer should be annealed at high temperature (150 °C used in this study, with higher temperatures not exceeding the melting point likely having a more significant impact, as suggested by the model) to pre-curl the device towards the base layer. Annealing the base Parylene layer increases stress in the layer, promoting curvature towards the base after the finished device is removed from the wafer due to shrinkage in the high-stress annealed Parylene.

The base Parylene layer should also be thicker than the top Parylene layer, keeping the total thickness requirement (⩽11.1 *µ*m) and the minimum layer thickness necessary for electrical insulation. An asymmetric PMP film stack promotes curvature by moving the high-stress metal layer away from the neutral plane of the device, with higher degrees of asymmetry producing more significant curvature (shown experimentally and with modeled conditions). In annealed or thermoformed devices, this results in the high stress metal layer dominating the bending moment, promoting curling towards the thicker Parylene layer.

If a highly asymmetric device is used, a thermoforming temperature of 200 °C should be used to take advantage of the stress imbalance in asymmetric devices. If a symmetric device is used, a thermoforming temperature of 100 °C is preferred to minimize shrinkage of the top Parylene layer (which negates the effects of the base Parylene anneal). High temperature annealing produces more significant changes from the unannealed device shape (due to the temperature dependence of annealed Parylene stress), while annealing time does not have as significant an impact.

## 7. Conclusion

In this study, annealing and thermoforming of thin film Parylene C strips and devices (with patterned thin film metal) were characterized and the results used to achieve thin film PMP helices down to 0.25 mm in diameter. Standard MEMS processes performed on soft polymers (such as Parylene) produced planar microfabricated devices, which were then formed into a helical shape via thermoforming. This process can also be applied to other 3D configurations, such as cylinders (for nerve cuff electrodes), spheres (for retinal electrode arrays), or cones (for tips of depth electrodes to interface with insertion stylets). In published work thus far, Parylene thermoforming has been used to produce devices with thermoformed functional regions (i.e. regions with thin film metal features) 1–5 mm in curvature diameter [1, 10–14] or non-functional regions (i.e. regions with bare Parylene) down to 0.25 mm in curvature diameter (with visible Parylene cracking) [1, 13].

PMP device curvature was also estimated using a basic mathematical model and material values from literature. The model, supported by experimental data, provides insight into design considerations for any desired geometry, whether the desired shape is flat or curved.

Overall, achieving a thermoformed Parylene device containing patterned thin film metal down to 0.25 mm in curvature diameter expands the design space for flexible 3D MEMS devices to allow for miniaturized electrodes, sensors, and other patterned metal features. More work is required to determine the effects of long term and/or *in vivo* use on device geometries (i.e. if moisture ingress, thermal cycling, or other environmental factors lead to changes in the device shape or function). Evaluation of additional helix diameters between 1.6 and 0.25 mm or below 0.25 mm would also provide valuable insight into the potential design space of thermoformed Parylene.

## Data Availability

All data that support the findings of this study are included within the article (and any supplementary files).
